# Lithotomy

**Published:** 1833

**Authors:** 


### X. Lithotomy.

The number of Lithotomists in the Western states, is rapidly increasing. Two new ones have lately made their *debut* in this city—Dr. Landon C. Rives, who operated on a boy 5 years of age, in Dayton, and Dr. McDowell, whose patient was a man of 60, in this city. Both operations were perfectly successful.

